# Diabetes-Prev Scale: validation of preventive measures for type 2 diabetes in inhabitants of Cajamarca, validation design

**DOI:** 10.15649/cuidarte.3797

**Published:** 2024-09-01

**Authors:** Orlando Linares-Vásquez, Yonathan Yoel Díaz-Dávila, José Ander Asenjo-Alarcón

**Affiliations:** 1 Licenciado en Enfermería. Universidad Nacional Autónoma de Chota, Chota, Cajamarca, Peru. E-mail: rolylv991@gmail.com Universidad Nacional Autónoma de Chota Universidad Nacional Autónoma de Chota Chota Cajamarca Peru rolylv991@gmail.com; 2 Licenciado en Enfermería. Universidad Nacional Autónoma de Chota, Chota, Cajamarca, Peru. E-mail: yonathandavila17@gmail.com Universidad Nacional Autónoma de Chota Universidad Nacional Autónoma de Chota Chota Cajamarca Peru yonathandavila17@gmail.com; 3 Doctor en Salud, Epidemiólogo. Facultad de Ciencias de la Salud, Universidad Nacional Autónoma de Chota, Chota, Cajamarca, Peru. E-mail: ander1213@hotmail.com Universidad Nacional Autónoma de Chota Facultad de Ciencias de la Salud Universidad Nacional Autónoma de Chota Chota Cajamarca Peru ander1213@hotmail.com

**Keywords:** Diabetes Mellitus, Type 2, Disease Prevention, Life Style, Health Risk Behaviors, Risk Groups, Diabetes Mellitus Tipo 2, Prevención de Enfermedades, Estilo de Vida, Conductas de Riesgo para la Salud, Grupos de Riesgo, Diabetes Mellitus Tipo 2, Prevenção de Doenças, Estilo de Vida, Comportamentos de Risco à Saúde, Grupos de Risco

## Abstract

**Introduction:**

Preventive measures effectively reduce the prevalence of diabetes; therefore, having valid instruments to assess them is a priority.

**Objective:**

To validate the content, construct, and reliability of the Diabetes-Prev Scale in the prevention of type 2 diabetes in the population of the Cajamarca region, Peru.

**Materials and Methods:**

Descriptive study with validation design. The scale was developed based on the recommendations of the American Diabetes Association and consists of 36 items validated by eight health and nutrition professionals. A survey pilot test was conducted with 385 adults living in the 13 provinces of Cajamarca. Construct validity was assessed using exploratory factor analysis and reliability using McDonald's œ.

**Results:**

The scale comprised 33 items divided into five dimensions: consumption of alcoholic beverages and cigarettes (11 items), harmful dietary and physical habits (6 items), beneficial eating and sleeping habits (5 items), food additives and body weight (6 items), and physical activity and hydration (5 items). Factor loadings were greater than 0.30, and the cumulative variance was 43.66%. The overall scale achieved a reliability of 0.88, making it an adequate and necessary instrument for assessing preventive measures for diabetes in the population.

**Discussion:**

The psychometric properties of instruments that assess various diabetes topics using equivalent statistical tests are also appropriate.

**Conclusions:**

The Diabetes-Prev Scale is a contextualized instrument for the three geographic regions of Peru, offering clear textual and content comprehension with a simple and brief application suitable for diverse populations.

## Resumen.

**Introducción:**

Las medidas preventivas son eficaces para reducir la prevalencia de diabetes, por ello contar con instrumentos válidos para su evaluación es prioritario.

**Objetivo:**

Validar por contenido, constructo y fiabilidad la escala Diabetes-Prev en medidas preventivas de diabetes tipo 2 en la población de la región Cajamarca, Perú.

**Materiales y Métodos:**

Estudio descriptivo, de diseño de validación. La escala se elaboró a partir de las recomendaciones de la Asociación Americana de Diabetes, constituida por 36 ítems, validados por ocho profesionales en salud y nutrición, se realizó una prueba piloto de encuesta con 385 personas mayores de edad, residentes en las 13 provincias de Cajamarca, la validez de constructo se obtuvo con el análisis factorial exploratorio y la fiabilidad con McDonald's œ.

**Resultados:**

La escala quedó conformada por 33 ítems distribuidos en 5 dimensiones: consumo de bebidas alcohólicas y cigarros (11 ítems), hábitos alimenticios y físicos perjudiciales (6 ítems), hábitos alimenticios y de sueño beneficiosos (5 ítems), aditivos alimentarios y peso corporal (6 ítems) y actividad física e hidratación (5 ítems), las cargas factoriales fueron mayores a 0,30 y la varianza acumulada de 43,66%. La escala global alcanzó una fiabilidad de 0,88 constituyéndose en un instrumento adecuado y necesario para evaluar las medidas preventivas de diabetes en la población.

**Discusión:**

Las propiedades psicométricas de instrumentos que valoran tópicos diversos de la diabetes, con pruebas estadísticas equivalentes, también son idóneos.

**Conclusión:**

La escala Diabetes-Prev es un instrumento contextualizado a las 3 regiones geográficas del Perú, con comprensión textual y de contenido y de aplicación sencilla y breve para poblaciones diversificadas.

## Resumo.

**Introdução:**

As medidas preventivas são eficazes na redução da prevalência da diabetes, pelo que ter instrumentos válidos para a sua avaliação é uma prioridade.

**Objetivo:**

Validar por conteúdo, construção e confiabilidade a escala Diabetes-Prev em medidas preventivas de diabetes tipo 2 na população da região de Cajamarca, Peru.

**Materiais e Métodos:**

Estudo descritivo, desenho de validação. A escala foi desenvolvida com base nas recomendações da American Diabetes Association, composta por 36 itens, validados por oito profissionais de saúde e nutrição. Foi realizado um teste piloto de pesquisa com 385 adultos, residentes nas 13 províncias de Cajamarca, obtendo-se validade de construto. com análise fatorial exploratória e confiabilidade com œ de McDonald.

**Resultados:**

A escala foi composta por 33 itens distribuídos em 5 dimensões: consumo de bebidas alcoólicas e cigarros (11 itens), hábitos alimentares e físicos prejudiciais (6 itens), hábitos alimentares e de sono benéficos (5 itens), aditivos alimentares e peso corpo (6 itens) e atividade física e hidratação (5 itens), as cargas fatoriais foram superiores a 0,30 e a variância acumulada foi de 43,66%. A escala global atingiu confiabilidade de 0,88, tornando-se um instrumento adequado e necessário para avaliar medidas preventivas do diabetes na população.

**Discussão:**

As propriedades psicométricas de instrumentos que avaliam diversos temas do diabetes, com testes estatísticos equivalentes, também são adequadas.

**Conclusão:**

A escala Diabetes-Prev é um instrumento contextualizado às 3 regiões geográficas do Peru, com compreensão textual e de conteúdo e aplicação simples e breve para populações diversificadas.

## Introduction

Chronic diseases have taken their place in today's society as the diseases with the highest incidence of morbidity and mortality in adults and older adults, including type 2 diabetes, which is appearing at an increasingly younger age and without timely diagnosis. Type 2 diabetes has been growing at a rapid and alarming rate for the past five years, and the trend is continuing. By 2021, the overall age-standardized prevalence reached 6.1%. Qatar had the highest prevalence in the world at 76.1%, mainly due to changes in the population's lifestyle, diet, physical activity, and daily habits. These factors have preliminarily led to an increase in obesity rates, which reached 52.2% worldwide in the same year, being one of the main causes associated with type 2 diabetes due to the pathophysiological mechanisms manifested^1^.

Early detection and control of risk factors such as overweight -which already affects two-thirds (60%) of the Norwegian population- and comorbidities for type 2 diabetes can delay the onset of the disease in individuals if prevention and promotion efforts are consistent and effective^2^^, ^^3^. Contemporary eating habits, marked by high consumption of processed foods, sugary drinks, and fast food (with 43.2% of Saudi adults preferring these options for convenience and ease of purchase), are reflected in the increase in body mass index, which accelerates the imbalance in blood glucose regulation mechanisms^4^. Likewise, physical inactivity or sedentary habits -as a result of increased body weight- and the frequent use of digital devices and vehicular transportation increase the detrimental effects at the functional level^5^.

Habits such as moderate or high consumption of alcoholic beverages are also associated with obesity and increased risk of type 2 diabetes, which almost doubles the likelihood of both due to the damage caused to the pancreas in the medium and long term, while at the same time predisposing to the development of cardiovascular events. In addition, people who consume alcohol underestimate the risks of it, which prevents them from assessing the consequences of excessive drinking^6^. Alcohol use is often accompanied by other habits, such as tobacco use, a significant risk factor for type 2 diabetes that nearly doubles the likelihood of developing the disease. This elevated risk can persist for up to 15 years after a person quits smoking^7^. The risk factors described are universal and present in all sociodemographic contexts.

These health-damaging practices are increasingly common in the population, and there is a lack of instruments to assess the preventive measures people take to avoid or delay the onset of type 2 diabetes. Current diabetes instruments measure aspects of the disease but not its prevention. These include the Diabetes Distress Scale (DDS) ^8^, which assesses patients' discomfort and anxiety about the disease; the Diabetes Self-Efficacy Scale^9^, which assesses patients' effectiveness in self managing the disease; and the 5-item Chinese version of the World Health Organization's Well Being Index (WHO-5-C) ^10^, which is used to identify signs of depression in patients with type 2 diabetes.

To assess the cognitive component of patients with type 2 diabetes, the Diabetes Knowledge Questionnaire^11^ is used to determine how much individuals know about the disease. Patient adherence can be measured using the General Medication Adherence Scale (GMAS) for patients with type 2 diabetes^12^. To measure patients' quality of life, the Diabetes Quality of Life Brief Clinical Inventory^13^ is available; patients' responsibility to maintain a preventive behavior to regulate their blood glucose levels can be assessed with the Hypoglycemia Awareness Questionnaire^14^. These instruments are used to assess issues related to type 2 diabetes and were used in the study as a reference for comparative analysis with the psychometric properties of the scale of preventive measures.

Estimating various aspects of type 2 diabetes is crucial for analyzing its progression and metabolic control as its burden increases over time. In Peru, the prevalence of this disease is 7%^15^ in people 25 years old and older, increasing with age in different regions of the country, including Cajamarca. However, it is more cost-effective, both economically and socially, as well as in terms of health care, to focus on the appropriate management of risk factors to promote a gradual change in the health behavior of the population. Therefore, the contribution with research inputs will guide the activities directed to these ends^16^.

Similarly, the diagnostic review conducted by practitioners on preventive measures for this type of disease will enable the quantification and qualification of educational resource investments aimed at prevention. This approach seeks to eliminate or reduce risk factors within the population to prevent the occurrence of type 2 diabetes. In this sense, the timely application of validated and contextualized instruments is very useful; therefore, the objective was to validate the Diabetes- Prev Scale in the prevention of type 2 diabetes in the population of the Cajamarca region of Peru.

## Materials and Methods

### Study design and sample

The research was descriptive because it determined the psychometric characteristics of the scale, and it was a validation design because it validated the scale's content, construct, and reliability. The study was conducted through a survey pilot test by sample strata (provinces) with 385 people aged 18 and older, residents of the 13 provinces of the Cajamarca region. The population was 971,105 people^17^, and the sample was calculated using a sampling proportion of 50%, a precision of 5%, and a confidence level of 95%. The sample was made up by stratified sampling, distributed as follows: Cajamarca, 102; Cajabamba, 20; Celendín, 22; Chota, 42; Contumazá, 8; Cutervo, 35; Hualgayoc, 23; Jaén, 53; San Ignacio, 35; San Marcos, 14; San Miguel, 14; San Pablo, 6, and Santa Cruz, 11. People with or without risk factors who were interested in learning about preventive measures for type 2 diabetes, had access to the Internet to complete the scale, and gave informed consent were included. On the other hand, people with no elementary education and those with a confirmed diagnosis of diabetes or cognitive impairment were excluded.

### Data collection technique and instrument

Data were collected through an asynchronous, self-administered, non-face-to-face survey. The instruments found in the literature serve to assess different clinical aspects of patients with type 2 diabetes^8^^, ^^14^, but there were no instruments available that refer to preventive measures for this disease. The scale was called the Diabetes-Prev Scale and was designed to assess preventive measures for type 2 diabetes in the population based on the American Diabetes Association recommendations for prevention (2023) ^18^. The preliminary version consisted of 36 items grouped in 5 dimensions, with response options: never or rarely = 1, sometimes = 2, often = 3, and almost always = 4. To ensure content validity, eight health and nutrition professionals were consulted and suggested that the name of two dimensions (3 and 4) be changed and two items (item 1 and item 11) be split, thus expanding the instrument to 38 items. In addition, they recommended that response options be differentiated by item type, with the following options: "less than two days a week" = 1, "two to three days a week" = 2, "four to six days a week" = 3, and "every day" = 4; and the options: "never" = 1, "sometimes" = 2, "often" = 3, and "always" = 4.

For construct validity, exploratory factor analysis was used. After its analysis, the scale consisted of 33 items distributed in 5 dimensions: consumption of alcoholic beverages and cigarettes (11 items), harmful dietary and physical habits (6 items), beneficial eating and sleeping habits (5 items), food additives and body weight (6 items), and physical activity and hydration (5 items). The instrument's reliability was optimal and was determined by the McDonald's œ coefficient. The percentile ranking allowed us to classify the overall score of the instrument.

### Data collection procedure

The data was collected through a form created and edited by the researchers using Google and shared on social media from September to November 2023. The form collected information on participants' demographic characteristics and frequency of application of preventive measures for type 2 diabetes. The simplicity of the form was evaluated with the participation of 20 individuals with characteristics similar to those of the sample, and appearance validation was determined. The form was then distributed to the study subjects; once the sample was completed according to the sample strata, the file containing the participants' responses was downloaded for data processing.

### Data analysis

Data were processed using the SPSS statistical package and stored in Mendeley Data^19^. Aiken's V was used to determine inter-rater agreement and was considered optimal when the value was > 0.75. The Kaiser-Meyer-Olkin (KMO) and Bartlett's sphericity tests were used to verify the assumptions, both of which were sufficient to apply exploratory factor analysis (EFA) and assess construct validity. EFA was performed using promax rotation and principal axis extraction methods. Reliability was determined using McDonald's œ, a test to calculate reliability when variables are ordinal and have three to four categories. All statistical tests were performed with a p-value < 0.05.

### Ethical considerations

Those who chose to participate in the study provided their informed consent electronically. During data collection, no identifying information was requested from the subjects, as the process was anonymous, and they were assigned a code according to the order in which they participated. The study adhered to the ethical principles of autonomy, justice, beneficence, and nonmaleficence, in line with the principles of online research^20^. The research was approved by a scientific committee of the Faculty of Health Sciences from Universidad Nacional Autónoma de Chota, through "Faculty Resolution N° 158-2023-FCCSS-UNACH/C.”

### Results

The study was conducted on 385 subjects aged 18 to 85 years (38.8 ± 14.1 years): 58.96% (n=227) were adults, 33.50% (n=129) were young people, and 51.94% (n=200) were women. Content validity was achieved with the satisfactory assessment of the items by the judges in terms of relevance, pertinence, sufficiency, adequacy and clarity, which was corroborated by an agreement of over 0.90 in Aiken's V on a global level (V= 0.973) and for each criterion assessed: adequacy (V= 0.976); sufficiency (V= 0.986); pertinence (V= 0.983); relevance V= 0.979, and clarity V= 0.944].

The verification of item assumptions was significant (p < 0.001) using Bartlett's test of sphericity and the KMO test for measures of sampling adequacy (MSA > 0.70). Item 7 (MSA= 0.630) and items 26, 33, 35, and 38 were discarded because they did not fit into any dimension (Table 1).

**Table 1:** KMO measures of sample adequacy by items

	Items	MSA
Global		0.900
Item 1	How often do you eat some of these fruits: apple, banana, pineapple, mango, peach, grapes, papaya, sapote, granadilla, lucuma, strawberries, blueberries, etc.?	0.852
Item 2	How often do you eat some of these vegetables: carrots, lettuce, cabbage, cauliflower, spinach, basil, chard, cucumbers, beet, radishes, Chinese onions, cilantro, parsley, etc.?	0.872
Item 3	How often do you eat stewed vegetables in your diet (peas, beans, lentils, lupini beans, chickpeas, fava beans, lima beans, etc.)?	0.801
Item 4	How often do you eat foods of animal origin (meat, fish, chicken, cheese, eggs, milk, etc.)?	0.823
Item 5	Do you drink coffee several times a day?	0.843
Item 6	How often do you use sweeteners such as light brown sugar, white sugar, chancaca (evaporated and solidified sugarcane juice), or sugarcane honey in higher-than- normal amounts to sweeten your beverages (> to 2 tablespoons for a 250 ml beverage)?	0.782
Item 7	How often do you include rice, potatoes, cassava, sweet potatoes, vitucas (tuber), ullucos (tuber), ocas (tuber), arracachas, wheat, barley, etc. in your diet?	0.630*
Item 8	How often do you drink processed beverages (soda, Frugos®, Cifrut™, Pulp™, Volt®, Gatorade®, Sporade™, Red Bull®, etc.)?	0.900
Item 9	How often do you eat foods containing flour (bread, biscuits, cakes, pies, alfajores (shortbread sandwich cookies), pastries, suspiro (meringue), nougat, etc.)?	0.894
Item 10	How often do you eat fried chicken, salchipapa (sausages and fries), salchipollo (chicken and fries), hamburgers, roasted chicken, BBQ wings, pancitas (grilled beef tripe), tripitas (grilled beef small intestine), anticuchos (grilled beef hearth)?	0.883
Item 11	How often do you drink 2 to 3 liters of pure water per day?	0.809
Item 12	At home, do you reuse cooking oil when making your food?	0.881
Item 13	At home, do you use animal fat when making your food?	0.899
Item 14	How often do you add extra salt to meals after they have already been served?	0.884
Item 15	How often do you consume snacks such as cookies, Chizitos® (extruded corn), Lay’s® (potato chips), Doritos® (tortilla chips), Los Cuates® (tortilla chips), Reyenito® (chocolate-filled cake), Chocman® (chocolate-covered cake), Vizzio® (chocolate-covered almonds), Princesa® (peanut cream-filled chocolate), Sublime® (chocolate bar), Cañonazo® (chocolate-covered bar), Tuyo™ (chocolate-covered wafer bar), gummy candies, chew gum, or Piqueo® (mixed chips)?	0.900
Item 16	Do you pay attention to maintaining an appropriate body weight?	0.858
Item 17	Do you walk, hike, jog, or run in your spare time?	0.822
Item 18	Does your job or daily routine involve spending much time sitting?	0.777
Item 19	Do you practice any sports like soccer, volleyball, basketball, cycling, or others?	0.864
Item 20	Do you use any transportation to get to your daily activities outside the home?	0.868
Items		MSA
Item 21	Do you get at least 150 minutes of regular physical activity weekly?	0.763
Item 22	How often do you drink alcoholic beverages, like beer, rum, wine, cañazo (sugarcane spirit), whisky, chicha de jora (fermented corn drink), Masato (fermented cassava drink), or others?	0.940
Item 23	At social gatherings, do you drink alcoholic beverages?	0.936
Item 24	At family gatherings, do you drink alcoholic beverages?	0.944
Item 25	At gatherings with friends, do you drink alcoholic beverages?	0.927
Item 26	Have any of your friends or relatives ever mentioned that you drink alcoholic beverages to excess?	0.842**
Item 27	For doing your daily activities, do you drink alcohol?	0.970
Item 28	After drinking alcoholic beverages, have you ever felt guilty or remorseful?	0.949
Item 29	How often do you smoke cigarettes?	0.938
Item 30	At social gatherings, do you smoke cigarettes?	0.930
Item 31	At family gatherings, do you smoke cigarettes?	0.947
Item 32	At gatherings with friends, do you smoke cigarettes?	0.933
Item 33	Have any of your friends or relatives ever mentioned that you smoke cigarettes to excess?	0.858**
Item 34	Are you exposed to cigarette smoke at home, at work, or in meetings?	0.968
Item 35	How often do you participate in family leisure activities, like playing video games, board games, watching TV, going to the movies, or similar activities?	0.759**
Item 36	In your free time, do you do activities like yoga, relaxation exercises, dancing, meditation, or prayer?	0.809
Item 37	Do you usually get 8 hours of sleep per day?	0.818
Item 38	Do you usually take a 15 to 30-minute rest after lunch?	0.832**

Test Kaiser-Meyer-Olkin (KMO) *Sample Adequacy Measures (SAM) < 0.70. **Did not fit into any dimension

Once the assumptions were verified, an exploratory factor analysis (EFA) was conducted to determine construct validity. The rotation and extraction methods were promax and principal axis factors, respectively. All items demonstrated factor loadings > 0.30. Parallel analysis allowed the number of factors to be determined. The cumulative variance of the 5 dimensions was 43.66% (dimension 1: 20.02%; dimension 2: 7.02%; dimension 3: 6.18%; dimension 4: 5.27% and dimension 5: 5.16%). The scale comprised 33 items divided into five dimensions: consumption of alcoholic beverages and cigarettes (11 items), harmful dietary and physical habits (6 items), beneficial eating and sleeping habits (5 items), food additives and body weight (6 items), and physical activity and hydration (5 items) (Table 2).

The 33-item Diabetes-Prev Scale demonstrated optimal overall reliability using McDonald's œ; reliability varied across dimensions (Table 3).

**Table 2:** Construct validity using exploratory factor analysis -EFA

Item	Factors				
	Dimension 1	Dimension 2	Dimension 3	Dimension 4	Dimension 5
Item 22	0.911				
Item 30	0.885				
Item 29	0.877				
Item 23	0.874				
Item 25	0.873				
Item 32	0.861				
Item 31	0.820				
Item 24	0.763				
Item 27	0.693				
Item 28	0.570				
Item 34	0.532				
Item 10		0.830			
Item 15		0.700			
Item 8		0.663			
Item 9		0.449			
Item 18		0.375			
Item 20		0.372			
Item 3			0.668		
Item 2			0.616		
Item 1			0.596		
Item 4			0.551		
Item 37			0.333		
Item 6				0.631	
Item 5				0.519	
Item 14				0.440	
Item 13				0.401	
Item 12				0.398	
Item 16				0.319	
Item 17					0.735
Item 21					0.701
Item 19					0.539
Item 36					0.325
Item 11					0.318

*Note: Promax rotation and principal axis factoring extraction*

**Table 3:** Diabetes-Prev Scale reliability

Scale	Mean ± SD	McDonald's œ
Total	1.636 ± 0.383	0.880
Dimension 1	0.926 ± 0.712	0.956
Dimension 2	2.041 ± 0.637	0.789
Dimension 3	2.394 ± 0.612	0.739
Dimension 4	1.793 ± 0.516	0.540
Dimension 5	1.769 ± 0.631	0.733

The classification of the instrument's total score was determined after verifying significant differences by sex and age group. For sex, the t-test showed a p-value of 0.000 with homogeneity of variances. For age group, ANOVA yielded a p-value of less than 0.005, and Scheffé's test resulted in a p-value greater than 0.472 for adults and older adults. The percentile ranking made it possible to classify the preventive measures for type 2 diabetes in the population. The scale scores range from 33 to 132 points. A percentile rank of > 71 indicates a favorable application of preventive measures; a rank of 21 to 70 indicates regular application; and a rank of < 20 indicates poor application (Table 4).

**Table 4:** Specific elements of the intervention.

Percentile	Sex			Age group*
	Male	Female	Young person*	Adult and older adult*
≤ 20	≤52 - 62	≤43 - 54	< 40 - 56	< 48 - 57
21 - 70	63 - 80	55 - 70	57 - 70	58 - 78
≥ 71	≥81	≥71	≥ 71	≥ 79

*Young person: 18 - 29years old; adult: 30 H 59years old; older adult: > 60 years old.

## Discussion

One of the most effective ways to reduce the burden of chronic diseases in the population is through the exhaustive, conscious, and sustained practice of preventive measures since the onset and persistence of chronic diseases are directly related to the convergence of multiple modifiable risk factors. Type 2 diabetes is no exception, as it is the disease that contributes most to the chronic disease burden.

The authors conceptualized 'type 2 diabetes preventive measures' as a set of conscious and continuous practices carried out by individuals to minimize or eliminate risk factors for type 2 diabetes.

When a comparative analysis was conducted with other instruments regarding their psychometric properties -though not their thematic content, as they assess different aspects of type 2 diabetes- it was found that these instruments also demonstrated ideal characteristics. The diabetes Self efficacy Scale^9^ was constructed from a satisfactory EFA with the extraction of a factor different from that obtained in our study, which is multidimensional. Similarly, the General Medication Adherence Scale in patients with type 2 diabetes^12^ and the Hypoglycemia Awareness Questionnaire^14^ achieved factorial validity with 3 and 4 factors, respectively. The difference with our study is the number of factors extracted due to the different extraction and rotation methods.

Regarding reliability, the Diabetes-Prev Scale is optimal. Compared with other instruments that assess different aspects of diabetes, it shows similar results in the reliability index, although not in the tests used. The Diabetes Distress Scale^8^ achieved a Cronbach's alpha of 0.92, the Diabetes Self-Efficacy Scale^9^ a Cronbach's alpha of 0.86, and the WHO-5-C Well-being Index^10^ a Cronbach's alpha of 0.88. The Diabetes Knowledge Questionnaire^11^ had a KR-20 of 0.77, the General Medication Adherence Scale^12^^)^ in patients with type 2 diabetes a Cronbach's alpha of 0.804, and the Brief Clinical Inventory of Quality of Life in Diabetes^13^ a Cronbach's alpha of 0.90. The Hypoglycemia Awareness Questionnaire^14^^)^ had reliability scores of 0.75 and 0.79, with Cronbach's alpha and McDonald's œ, respectively. The differences in the statistics used to estimate reliability depend on the number of response options in each item and its measurement scale.

The EFA distributed the items across 5 dimensions, resulting in a multidimensional scale based on factor loadings > 0.30. Dimension 1: "Consumption of alcoholic beverages and cigarettes," consisting of 11 items, refers to the frequency and circumstances under which individuals consume these products. The items include frequency of consumption of alcoholic beverages (beer, rum, wine, Cañazo, whisky, Chicha de Jora, Masato, and others), consumption of alcoholic beverages in social gatherings, with family, friends, or in daily activities, and feelings of guilt or remorse after drinking alcohol. They also include the frequency of cigarette use, cigarette use in social gatherings, with family or friends, and exposure to cigarette smoke.

In Dimension 1, item 22 is the one that best explains the construct, as it has the highest factor loading of 0.911 and refers to the frequency of consumption of alcoholic beverages based on their type. Whether alcohol consumption is a risk factor for developing type 2 diabetes will depend on the concentration of alcohol in the beverages, the quantity consumed, the timing of consumption, the intervals between consumption, and whether the stomach is empty. Empty stomach conditions can initially cause hypoglycemic spikes and, over time, may disrupt glucose regulation mechanisms, potentially increasing the risk of new-onset type 2 diabetes^6^^, ^^21^.

As a preventive measure, abstinence from alcohol and cigarettes is beneficial to individual health because it reduces organic damage to individuals in the medium and long term and to public health because it contributes to reducing the incidence of type 2 diabetes, provided that abstinence is sustained and continuous^22^. Therefore, health promotion strategies to modify this ingrained behavior in the population should be addressed by nursing professionals through an exhaustive analysis of the intervening variables for a comprehensive approach.

Dimension 2, 'harmful dietary and physical habits,' consists of 6 items that assess the frequency of consuming products with a high glycemic index and engaging in activities that involve minimal physical exertion. The items include frequency of consumption of processed beverages, flour-based foods, fast foods, and sweets; daily sitting time, and use of transportation for activities outside the home. Item 10 best explains the construct with a factor loading of 0.830 and refers to the frequency of fast-food consumption. If this practice continues, it will produce a sudden and accelerated increase in blood glucose, which alters the normal rhythm the regulatory mechanisms^4^.

Harmful eating habits, such as the consumption of ultra-processed foods or foods with a high glycemic index, should be avoided and replaced with more frequent consumption of fruits, vegetables, and foods containing fiber and vegetable oils. Choosing a healthy diet is one of the most successful ways to increase survival and improve quality of life. Similarly, if this practice is combined with daily activities that maintain an adequate energy balance, obesity rates will be reduced, as will those of type 2 diabetes^23^. In this sense, health education aimed at generating knowledge and raising awareness among the population is a crucial focus that should be included in the agenda of community and public health nurses.

Dimension 3, "beneficial eating and sleeping habits," consists of 5 items that refer to the frequency of consuming foods beneficial to the body and adequate sleep time. The items in this dimension include the frequency of consuming fruits, vegetables, vegetable stews, foods of animal origin, and the amount of time dedicated to sleep. Item 3 is the item that contributes the most to this construct, with a factor loading of 0.668. It refers to the frequency of consuming vegetable stews in the diet, which should be eaten regularly due to their low glycemic index and slow absorption.

Daily consumption of fruits and vegetables, as well as foods that provide nutrients to the body, is very beneficial because of their association with reducing the risk of several non-communicable diseases, including type 2 diabetes. It also reduces the risk of cardiovascular disease and stroke, so these habits should be promoted in the population. If we add to this the recovery of the body and brain through restful sleep, people will have additional benefits against diseases and improvements in their quality of life^24^. While not all people are in the same situation regarding eating healthily or including certain foods in their daily diet, with the nurses' advice, they can take advantage of the nutritionally equivalent products in their area of residence.

Dimension 4,"Food additives and body weight," consists of 6 items that refer to incorporating products in food preparation or consumption and maintaining an appropriate body weight. The items consist of frequency of coffee consumption and use of sweeteners, reusing oil to prepare food, using animal fat to prepare food, adding salt to already prepared meals, and being aware of adequate body weight. Item 6 has the highest factor loading at 0.631 and best explains the construct related to the frequent use of sweeteners like sugar, chancaca (evaporated and solidified sugarcane juice), or honey in larger- than-normal amounts, leading to blood glucose overload and fluctuations in insulin release by the pancreas.

Using additives in the diet can be detrimental to health, as it is associated with an increased risk of developing type 2 diabetes and with an increase in body weight due to continuous blood glucose overload and damage to the vascular endothelium by indirect mechanisms. For this reason, their consumption should be reduced through educational campaigns that raise awareness about the harm these products can cause to the body^25^^, ^^26^. Therefore, the promotion and prevention activities carried out by nurses must be based on a prior diagnosis of all the risk factors to which individuals are exposed.

Dimension 5, "Physical activity and hydration," includes 5 items that address recreational and relaxation activities, as well as adequate water intake. The items include the frequency of drinking plain water, walking, strolling, jogging, playing sports, engaging in regular physical activity, and participating in relaxation activities. Item 17 is the item that best explains the construct with a factor loading of 0.735 and is related to walking, strolling, jogging, or running, practices that provide multiple benefits to the body by stimulating the proper use of glucose and removing adipose tissue deposits.

Physical activity is a preventive measure that complements the previous ones and it should be recommended at the different levels of health care, as it favors glucose control at adequate levels and better utilization of glucose by the cells. In this way, it can contribute to reducing the prevalence of type 2 diabetes in the population^27^^, ^^28^. Physical activity should be included in nurses' preventive regimens to complete the comprehensive preventive package for diabetes risk factors and related conditions.

The Diabetes-Prev Scale's reliability is optimal, which indicates the instrument's efficient internal consistency and guarantees favorable results when used.

The limitations of the study are as follows: The virtual application of the scale may have created certain response biases in the participants, but it was the only way to collect the information due to the geographical distribution of the participants in the 13 provinces of the Cajamarca region, which was not feasible for the researchers to do in person. This limitation was overcome by administering the instrument to a representative group beforehand to assess its simplicity and by working with a probability sample for the pilot test. On the other hand, the response rate of the participants was high (97.00%), which implies a minimal selection bias. Finally, the absence ofa gold standard instrument to compare the scale's psychometric properties limits our ability to assess its effectiveness in evaluating preventive measures for type 2 diabetes; however, its benefits are highly favorable and enable us to obtain conclusive results.

## Conclusion

The Diabetes-Prev Scale is a multidimensional scale with suitable characteristics in terms of content validity, construct validity, and reliability, making it suitable for use in different populations to assess preventive measures implementation for type 2 diabetes. It is understandable and simple, and its use can be extended to make a differentiated assessment of preventive measures for individuals, according to their educational level, marital status, income, and exposure to risk factors, in order to program more targeted interventions.

As the first of its kind, the Diabetes-Prev Scale is a suitable, inexpensive, affordable, and necessary instrument for primary health care, offering an overview of the population's current engagement in protective healthy practices. This serves as a starting point for nurses who are responsible for prevention and health promotion activities to organize, plan, schedule, implement, and monitor a comprehensive preventive care package for diabetes risk factors and related conditions.
